# Mesoscopic Multimodal Imaging Provides New Insight to Tumor Tissue Evaluation: An Example of Macrophage Imaging of Hepatic Tumor using Organosilica Nanoparticles

**DOI:** 10.1038/s41598-017-04043-7

**Published:** 2017-06-21

**Authors:** Michihiro Nakamura, Koichiro Hayashi, Hitoshi Kubo, Masafumi Harada, Keisuke Izumi, Yoshihiro Tsuruo, Toshinobu Yogo

**Affiliations:** 1 Department of Organ Anatomy and Nanomedicine, Yamaguchi University Graduate School of Medicine, 1-1-1 Minami-Kogushi, Ube Yamaguchi, 755-8505 Japan; 2 Division of Materials Research, Institute of Materials and Systems for Sustainability, Nagoya University, Fro-cho, Chikusa-ku Nagoya, 464-8603 Japan; 3 Preparing Section for New Faculty of Medical Science, Advanced Clinical Research Center, Fukushima Global Medical Science Center, Fukushima Medical University, 1 Hikariga-oka, Fukushima, 960-1295 Japan; 4 Department of Radiology, Tokushima University Graduate School of Medical Sciences, 3-18-15 Kuramoto, Tokushima, 770-8503 Japan; 5 Department of Molecular and Environmental Pathology, Tokushima University Graduate School of Medical Sciences, 3-18-15 Kuramoto, Tokushima, 770-8503 Japan; 6 Department of Anatomy and Cell Biology, Tokushima University Graduate School of Medical Sciences, 3-18-15 Kuramoto, Tokushima, 770-8503 Japan

## Abstract

Multimodal imaging using novel multifunctional nanoparticles provides new approach to biomedical field. Thiol-organosilica nanoparticles containing iron oxide magnetic nanoparticles (MNPs) and rhodamine B (thiol OS-MNP/Rho) were applied to multimodal imaging of hepatic tumor of Long−Evans Cinnamon (LEC) rat. The magnetic resonance imaging (MRI) of LEC rats revealed tumors in the liver clearly and semi-quantitatively due to a labeling of macrophages in liver. The fluorescent imaging (FI) showed abnormal fluorescent patterns of the liver at the mesoscopic level that was between macroscopic and microscopic level. We performed correlation analysis between optical imaging including FI and MRI. We found that the labeled macrophages located specific area in the tumor tissue and influenced the tumor size on MRI. In addition histological observation showed the labeled macrophages related specific tissue in the pathological region. We demonstrated a new approach to evaluate tumor tissue at the macroscopic and microscopic level as well as mesoscopic level using multimodal imaging.

## Introduction

Multimodal imaging combines two or more imaging modalities into one system and is an important approach for advanced imaging studies^1–10^. Fluorescent imaging (FI) and magnetic resonance imaging (MRI) have unique relations because their advantages and disadvantages compensate each other. MRI has various contrasts, such as T1 and T2-weighted, and has several advantages: higher spatial resolution and the fact that physiological, molecular, and anatomical information can be extracted simultaneously. However, MRI has relatively low sensitivity and a long scan time and large quantity of probe may be needed. FI has already been developed for *in vitro* and *in vivo* applications. Notable advantages of FI are high temporal resolution, high sensitivity, multichannel imaging using multiple fluorescent probes, and relatively low-cost. However, FI *in vivo* is not quantitative, and the image information is surface-weighted. A fusion of the advantages of MRI and FI and their compensation of their disadvantages enables the creation of a novel imaging system.

Recently various types of multimodal imaging nanoparticles (NPs) have been investigated and applied for MRI and FI of hepatic tumor^11–13^. Multimodal imaging NPs containing magnetic nanoparticles (MNPs) and fluorescent nanoparticles (FNPs) can be applied to T2-weighted MRI and FI. In the liver, NPs are rapidly taken up by specialized macrophages, Kupffer cells. Imaging of macrophages using their naturally high endocytosis activity of NPs is called “macrophage imaging”^14^. In the hepatic tumor, MNPs increase the contrast between healthy and tumor tissue lacking Kupffer cells, because the healthy tissue decreases the signal. Resovist consisting superparamagnetic iron oxide nanoparticles coated with carboxydextran has been approved for clinical diagnosis of hepatic tumor using MRI. Therefore “macrophage imaging” of hepatic tumor is very useful and practical clinical approach. Multimodal imaging of haptic tumor is also investigated and reported^11–13^. However, importance of multimodal “macrophage imaging” to evaluate hepatic tumor tissue has not been reported very well.

**Figure 1:**
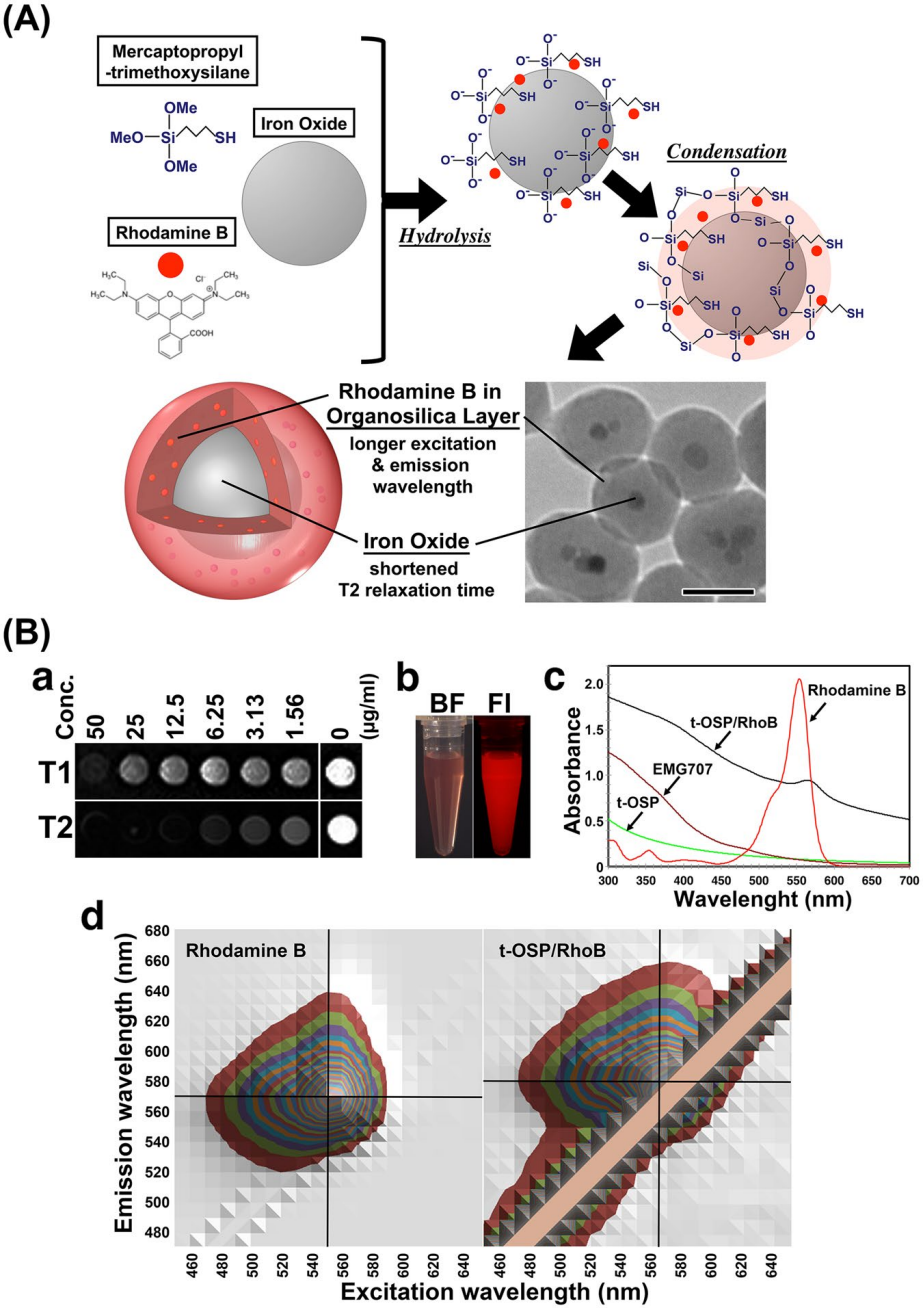
Preparation and characterization of thiol-OS-MNP/Rho. (**A**) Scheme of the formation and electron microscopic finding of thiol-OS-MNP/Rho. (**B**) Panel a shows the T1 and T2 weighted images that were obtained with spin echo and turbo spin echo seaquence, respectively. Acquisition patameters were as belows; (T1 weighted image) TR = 300 ms, TE = 10 ms, 3 mm thickness, FOV = 240 × 144 mm^2^, matrix = 256 × 108, NEX = 8, (T2 weighted image) TR = 5000 ms, TE = 89 ms, 3 mm thickness, FOV = 240 × 142 mm^2^, matrix = 448 × 160, NEX = 4. Panel b showed findings of bright field imaging (BF) and fluorescent imaging (FI). (c) Absorbance spectra of t-OSP/RhoB, rhodamine B, EMG707, and thiol organosilica particles without rhodamine B nor EMG707. (d) Comprehensive 3-dimentional analysis of the excitation, emission, and fluorescence intensities for rhodamine B and t-OS-MNP/Rho.

We are developing novel types of silica NPs prepared from a single organosilicate and referred as organosilica NPs^15–19^. These organosilica NPs are both structurally and functionally different from typical (inorgano)silica particles prepared from tetraethoxyorthosilicate (TEOS). Organosilica NPs can contain not only functional molecules such as fluorescent dye^20, 21^ but also nanomaterials^22, 23^. Functionalized organosilica particles were applied for biomedical research such as imaging *in vitro* ^20, 22^ and *in vivo* ^21–25^, and single cell analysis^26, 27^. In addition functional molecules and nanomaterials could show new function in organosilica nanoparticles^21–23^. Recently we reported that organosilica NPs containing MNPs and rhodamine B (thiol-OS-MNP/Rho) revealed shortened T2 relaxation time of MNPs and longer excitation wavelength of rhodamine B^23^ (Fig. 1). In this paper, we report the first usefulness of multimodal imaging of pathological liver in Long−Evans Cinnamon (LEC) rats with a fusion of optical imaging including FI and T1- and T2-weighted MRI using thiol-OS-MNP/Rho. These imaging provided seamless and sequential “identical” multimodal imaging of tumor tissue from at macroscopic to microscopic level including at their intermediate level (mesoscopic imaging). In addition, we demonstrated that “differential” multimodal imaging of hepatic tumor between optical imaging including FI and MRI was also very important to evaluate and understand tumor tissue. We discuss a new insight of hepatic tumor tissue using multimodal imaging.

## Results and Discussion

### Preparation and characterization of thiol-OS-MNP/Rho

Thiol-OS-MNP/Rho (diameter 88 nm) was prepared by a one-step, one-pot synthesis using 3-mercaptopropyltrimethoxysilane (MPMS) as the single silica source as reported previously^23^. The formation schema of the thiol-OS-MNP/Rho was shown in Fig. 1A. Thiol organosilica layer was formed with rhodamine B after both the hydrolysis and condensation reaction on the surface of EMG707. Thiol-OS-MNP/Rho were monodisperse and contained from one to a few MNPs per particle as shown in electron microscopic picture in Fig. 1A.

The T1- and T2-weighted MRI of phantom containing various concentrations of thiol-OS-MNP/Rho are shown in Fig. 1Ba. Relaxivities (R1 and R2) of thiol-OS-MNP/Rho of were 3.07 × 10^–3^ and 2.97 × 10^–8^ msec^−1^·μg·ml^−1^ respectively. A solution containing 0.1 mg/ml of thiol-OS-MNP/Rho showed fluorescence clearly on fluorescent microscopy (Fig. 1Bb). The absorbance spectra of thiol-OS-MNP/Rho showed a peak at 563 nm, while rhodamine B appears at 554 nm (Fig. 1Bc). In addition thiol-OS-MNP/Rho showed broad absorbance even above 600 nm. Comprehensive 3-dimensional analysis of the excitation, emission, and fluorescence intensities of rhodamine B solution and thiol-OS-MNP/Rho (Fig. 1Bd) were performed. A fluorescence profile of the thiol-OS-MNP/Rho was different from that of rhodamine B solution. The rhodamine B solution had excitation/emission maxima of 550/570 nm. The excitation/emission maxima of the thiol-OS-MNP/Rho were 560/580 nm. These results demonstrated that the shift of the absorbance and fluorescence peaks to longer wavelengths were observed. Specific emission with excitation above 600 nm was not observed. These results suggested that the fluorescent property of rhodamine B was altered in organosilica layer. The organosilica layer has an organosilica network connected by a mercaptopropyl chain and is organic/inorganic hybrid structures. Various interactions, such as an energy and electron transfer, occur between rhodamine and the organosilica structure. Previously, we reported unique changes to the fluorescence properties of QDs^22^ and propidium iodide^21^ upon incorporation within the thiol-organosilica particle. Their fluorescence profile had an extended range of excitation wavelengths toward longer wavelengths. The mechanisms by which rhodamine B as well as other fluorescent molecules in the organosilica layer or in organosilica particles alter the fluorescence properties are under investigation.

### *In vivo* MRI of LEC rat injected thiol-OS-MNP/Rho

**Figure 2:**
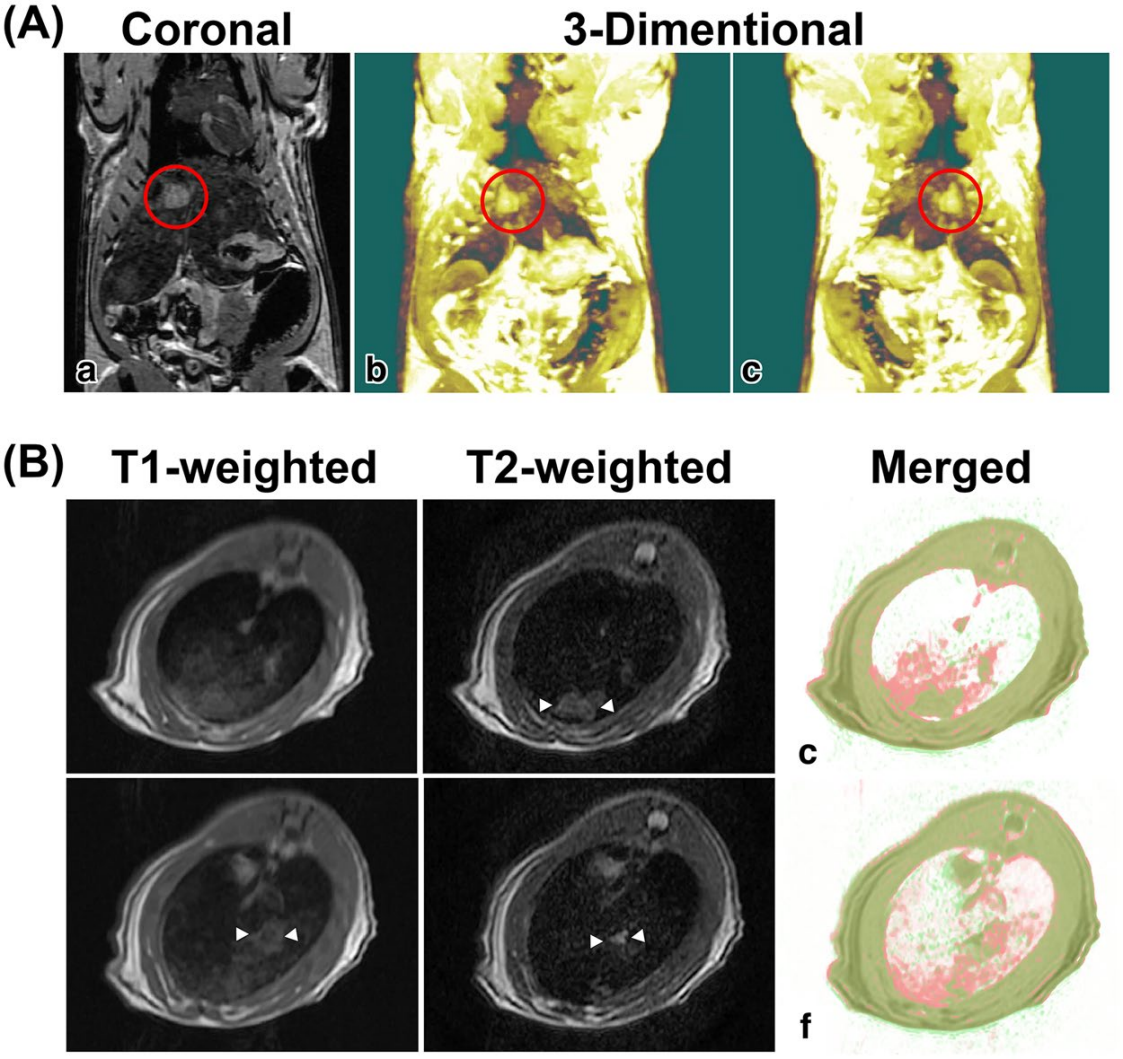
*In vivo* MRI of LEC rats injected thiol-OS-MNP/Rho. LEC rats (57 weeks) were injected intravenously with 0.9 mL of a solution containing 10 mg/mL of thiol-OS-MNP/Rho. After 1 day, rat was examined on MRI. (**A**) Coronal view (a), 3-dimensional images (b and c) in the 3D-SPGA MRI revealed a large tumor (marked with a red circle) and some small nodules in the liver. (**B**) The T2-weighted MRI (b), but not T1-weighted MRI could detect a nodular high intensity area (marked white triangles) at the same transverse level. At another level, the T1-weighted (c) and T2-weighted (d) MRI showed large nodular area with a moderate intensity and a small nodular area with high intensity, respectively. The T1- and T2-weighted images (a,b and d,e) were merged (c and f, respectively). Some areas in the T1-weighted (red) were identical with the T2-weighted (green) in liver.

An LEC rat of approximately 13 months old was injected with thiol-OS-MNP/Rho intravenously. The LEC rat has been established from a closed colony of Long-Evans rats^28^. LEC rats develop spontaneous hepatitis approximately 4 months after birth, followed by death in 50% of rats due to fulminant hepatitis^29, 30^. The recovered rat from the fulminant hepatitis exhibited chronic hepatitis and cholangiofibrosis and subsequent high rate of liver cancer^31^. We performed multimodal imaging of LEC rat injected with thiol-OS-MNP/Rho. The coronal view (Fig. 2Aa), 3-dimensional images (Fig. 2Ab and c and Supporting information movie S1) in the 3-dimensional spoiled gradient recalled acquisition (3D-SPGR) revealed a large tumor (marker with red circle) and some small nodules in the liver. The thiol-OS-MNP/Rho can enhance and detect the tumor and abnormalities of LEC rat liver in MRI clearly and non-invasively. These higher spatial resolutions could assist an indication to find the pathological region in FI.

As shown in Fig. 2B, and Supporting information movie S2a and S2b for transverse findings of T1- and T2-weighted MRI respectively, the T1- and T2-weighted MRI of the LEC rat liver revealed abnormal findings, but the findings were different from each other. On T2-weighted MRI, the liver showed a nodular high intensity area (Fig. 2Bb), but there was diffuse moderate intensity, not a nodular area in the T1-weighted MRI (Fig. 2Ba) at the same level of transverse view. At another level, the T1-weighted MRI showed a large nodular area with a moderate intensity (Fig. 2Bd) and a small nodular area with high intensity in the T2-weighted MRI (Fig. 2Be). These moderate intensity areas in the T1-weighted were merged with those of the T2-weighted to compare their distribution (Fig. 2Bc, f). Some areas in the T1-weighted (red) were identical with the T2-weighted (green), but other larger areas were not. As reported previously^23^, thiol-OS-MNP/Rho enhanced shortening of the T2 relaxation times, but not of the T1 relaxation times. Therefore it was possible that difference between T1-weighted and T2-weighted findings could be enhanced. The organosilica layer contains proton molecules in the propyl residue and many diamagnetic elements, such as carbon, sulfur, and silicon. These diamagnetic elements might enhance shortening of the T2 relaxation times, but not of the T1 relaxation times of thiol-OS-MNP/Rho. There were three patterns of MRI signal in the LEC rat liver, white, red, and green merged with red in merged image (Fig. 2Bc and f). We classified the white areas as containing a high amount of labeled cells at a similar level with normal liver. Red areas contained labeled cells lower than that of white areas, and green merged with red areas contained lower levels of cells than that of the red area. The red areas and green merged with red areas indicated pathological areas. Correlation analysis between the T1- and T2-weighted images provided semi-quantitative information about the distribution of labeled cells in pathological liver. Multimodal imaging with FI could further information about pathological areas as described below. In addition to multimodal imaging, a combination with physicochemical analysis such as inductively coupled plasma mass spectrometry (ICP-MS) for organs, tissues, and pathological areas would provide more quantitative information. Further studies are required for integrative evaluation system for the biodistribution of nanoparticles.

### Mesoscopic multimodal imaging of pathological liver of LEC rats

**Figure 3:**
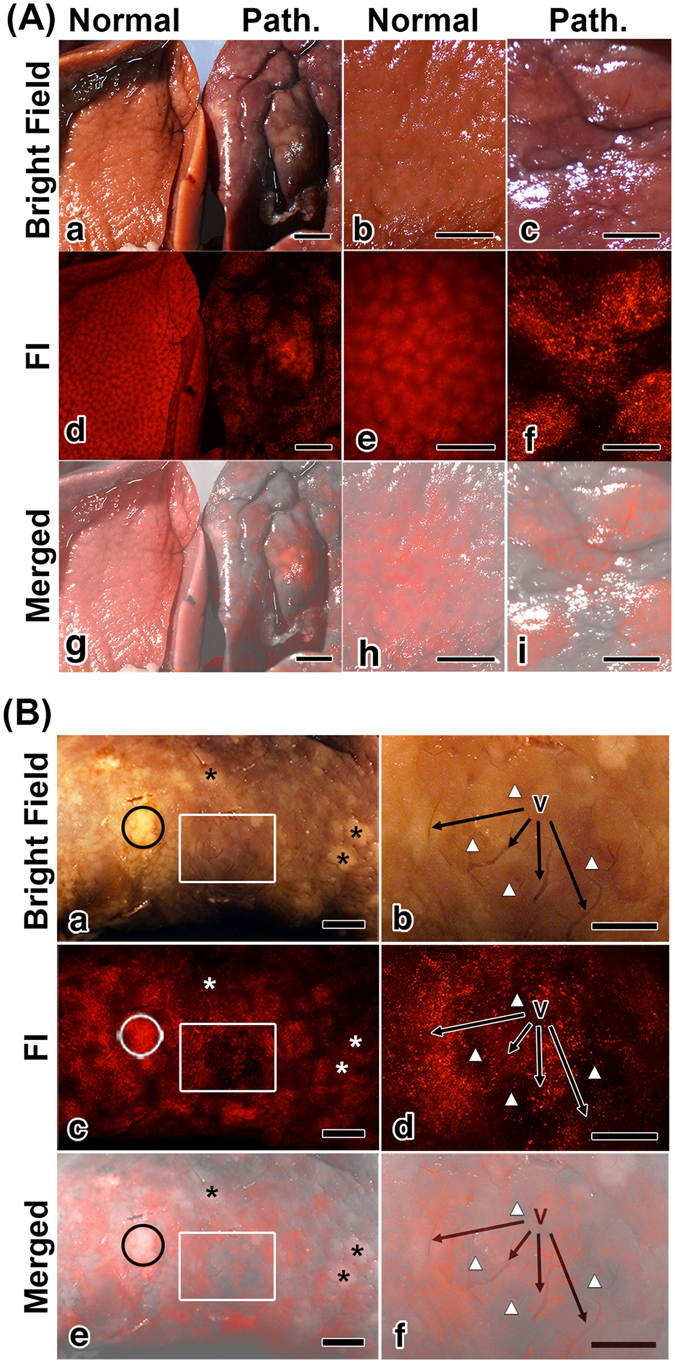
Mesoscopic FI of a pathological liver of LEC rat. (**A**) Normal (a,b,e) and pathologic (Path.) (a,c,f) liver were observed on bright field (a–c) and FI (d–f). Normal liver showed a fluorescent reticular hepatic lobular pattern (d and e), but the pathologic liver did not (d and f). Panels (g),(h) and (i) are merged images of (a) and (d),(b) and (e) and (c) and (f), respectively. Scale bars:  (a,d,g) 2 mm, (b,c,e,f,i) 1 mm. (**B**) The pathologic liver was observed with higher magnification. There were white nodules marked with asterisks and open circle in the bright field image (a). The surface of the pathologic liver showed a heterogeneous distribution of labeled cells in the FI (c). Panels (e) is merged images of (a) and (c). Areas indicated by a white box in panel (a,c,e) were observed with high magnification. Some vessels (V) were observed in the bright field image (b). Areas with a lower distribution of labeled cells (white triangle) were observed in FI (d). The merged image of (b) and (d) revealed that there were higher numbers of labeled cells around the vessels (V) than far from vessels (white triangle) (f). Scale bars:  (a,c,e) 2 mm, (b,d,f) 1 mm.

The liver of LEC rats injected with thiol-OS-MNP/Rho was removed and observed by multipurpose zoom fluorescence microscopy. As shown in Fig. 3A, mesoscopic FI demonstrated predominant different findings between normal and pathological livers. Normal liver showed a fluorescent reticular hepatic lobular pattern (Fig. 3Ad and e) as the similar finding to that of normal mouse liver^23^. In the normal liver, there are liver lobules consisting of the liver cells (hepatocytes). The hepatocytes form cellular plates, and the space between these plates contains capillaries, the liver sinusoids. The liver sinusoids contain not only endothelial cells but also macrophages called as Kupffer cells. The Kupffer cells were located in the peripheral region of the liver lobule predominantly. Therefore the polygonal structures of the liver lobules in mesoscopic FI of LEC rats were enhanced by thiol-OS-MNP/Rho in Kupffer cells. In this study using rats, FI showed reticular patterns based on the normal histological structure (Fig. 3Ad and e) too, but pathologic liver did not show clear reticular lobular patterns (Fig. 3Ad and f). In addition pathologic liver showed lower general fluorescent intensity compared with normal liver (Fig. 3Ad). These findings indicated an alteration of structural change of liver tissue and a decrease of the number and/or the uptake of Kupffer cells in at least the surface area of liver. There were heterogeneous distributions of labeled cells without clear reticular lobular patterns (Fig. 3Af) as compared with that of normal liver (Fig. 3Ae). The distribution of labeled cells of pathologic liver showed larger clusters than that of normal liver lobule. Any relation between these large clusters and specific findings, such as protrusions or dents on the surface, were not found (Fig. 3Ai).

Figure 3B shows the surface of pathologic liver with higher magnification. There were white nodules marked with asterisks and open circle on the surface in the bright field image (Fig. 3Ba). The surface of the pathologic liver showed a heterogeneous distribution of labeled cells in FI (Fig. 3Bc). The areas of white nodules indicated by open circle showed a higher number of fluorescence labeled cells, but the areas marked with asterisks had a lower number, as shown in the merged image (Fig. 3Be). These results demonstrated that the findings of white nodules in the bright field could not relate the distribution of labeled cells in FI. There were differences between the bright field and fluorescent imaging. The nodules marked with asterisks would show high contrast in the MRI because of labeled cell decrease. But the nodule marked with open circle would show normal contrast on MRI because of a lot of labeled cell. It is possible that these “differential” multimodal imaging would provide further understanding the relation between macrophages and tumor progression.

An area indicated in a white box showed some vessels on the bright field image (Fig. 3Bb) and indicated an area with a lower distribution of labeled cells compared with those of its surrounding in FI (Fig. 3Bd). The merged image (Fig. 3Bf) revealed that there were higher numbers of labeled cells around vessels than far from vessels (white triangle). These findings indicated that the distributions of labeled cells were related with the vessels in this area. As shown in the movie (Supporting information movie S3) of Fig. 3B, the labeled cells in the liver were detected using real time mesoscopic FI. This movie demonstrates a real time detection of various pathological findings from low to high magnifications. These findings revealed high possibilities to develop mesoscopic FI-guided surgical treatment using fluorescent NPs. The mesoscopic FI of pathological liver of LEC rats revealed predominant differences from that of normal liver. This is the first report describing pathological mesoscopic FI of liver using FNPs. The mesoscopic FI of the surface fluorescence patterns and distributions of labeled cells of various diseases, and their alterations with the progress of diseases, would be useful for “macrophage imaging”. Mesoscopic FI is a novel imaging approach to detect and to evaluate pathological regions. In addition, mesoscopic FI would enable a correlation of the macroscopic findings in MRI and the microscopic findings of microscopy. However further studies to understand these findings are required, mesoscopic FI would be a novel diagnostic technique and an assistive technology for surgery and tissue-specific treatment.

**Figure 4:**
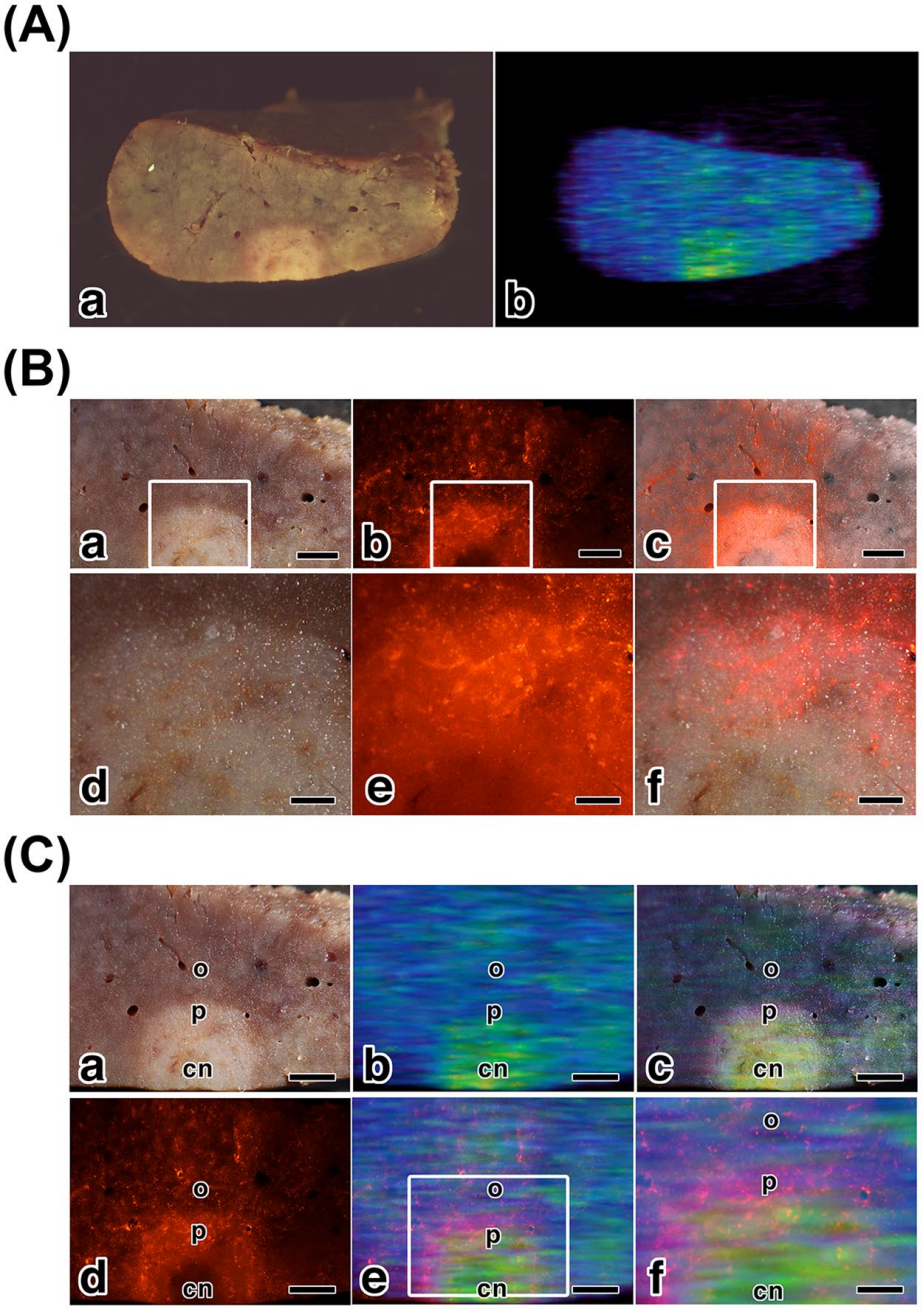
Mesoscopic multimodal imaging of a sectioned liver of LEC rat. (**A**) The section of the pathologic liver showed a white nodule in the bright field image (a), and in the T2-weighted MRI (b). (**B**) The peripheral area (p) of the white nodule were observed on bright field (a and d) and on FI (b and e). The merged images (c and f) showed differences in the number of labeled cells among outside (o), peripheral (p), and central (cn) area of the white nodule. Scale bars:  (a–c) 1 mm, (d–f) 0.25 mm. (**C**) Correlation analysis between the FI and MRI findings of the sectioned pathological liver. Compared with the bright field image (a), the findings of the MRI (b) and the merged image (c) showed that the size of the nodular area in the MRI was smaller than that of the bright field image. The merged images of the MRI and FI demonstrated the MRI signals were surrounded by FI signal (e and f). Scale bars:  (a–e) 1 mm, (f) 0.5 mm.

Next, we observed the sectioned pathological liver of LEC rat by using FI and MRI to evaluate the correlation between these two modalities. As shown in Fig. 4Aa, the section of the pathologic liver showed a white nodule inside a bright field image. The white nodule was detected in the T2-weighted MRI as a round signal (Fig. 4Ab, and the Supporting information movie 3). These findings demonstrated that the macroscopic multimodal imaging of MRI and bright field image were identical. As shown in Fig. 4B, the white nodule and its surrounding area were observed by FI. The peripheral area of the white nodule showed a larger number of labeled cells than those of the outside area (Fig. 4Bb). The central area of the white nodule did not show many labeled cells (Fig. 4Bb and e). The merged images (Fig. 4Bc and f) showed differences in the number of labeled cells among the outside, peripheral region, and central region of the white nodule. The section of pathologic liver showed peripheral and circular distributions of the labeled cells in the tumor. The region specific localization of the macrophages in the white nodule was detected using mesoscopic FI. These findings demonstrated that mesoscopic FI of the section provided further information about the distribution of the macrophages in tumors. Macrophages are associated with various pathological changes, such as inflammatory diseases and tumors^32–36^. In tumor biology, macrophages that infiltrate the tumor stroma are called tumor-associated macrophages (TAMs). Several papers reported that TAMs promote cancer cell growth, invasion, metastasis, angiogenesis, and suppression of antitumor immunity. Furthermore an increased number of TAMs is frequently correlated with angiogenesis, metastasis, and poor prognosis^37–39^. Therefore, a relation between macrophages and tumors is very important. The mesoscopic FI of pathological liver has high potential to understand the importance of macrophage localization in tumors and its relation with tumor progression.

We performed correlation analysis between optical imaging and MRI mesoscopic findings of sectioned pathological liver *ex vivo* (Fig. 4C). Compared with the size of the white nodule in the bright field image (Fig. 4Ca), that of the signal of the MRI (Fig. 4Cb) was smaller. The merged image (Fig. 4Cc) also revealed a size difference between the findings of bright field imaging and that of MRI. They display “differential” mesoscopic multimodal imaging. The FI (Fig. 4Cd) showed a peripheral and circular distribution of labeled cells in the nodule of the bright field image. The merged images of the MRI and FI (Fig. 4Ce and f) demonstrated that the MRI signals were surrounded by FI signals. These findings indicated that the peripheral and circular distribution of the labeled cells in the white nodule made the signal on the MRI smaller than that in the bright field image. The “differential” mesoscopic multimodal imaging is important for understanding tumor size evaluation in MRI.

### Histological FI of the Pathological Liver of the LEC Rats

**Figure 5:**
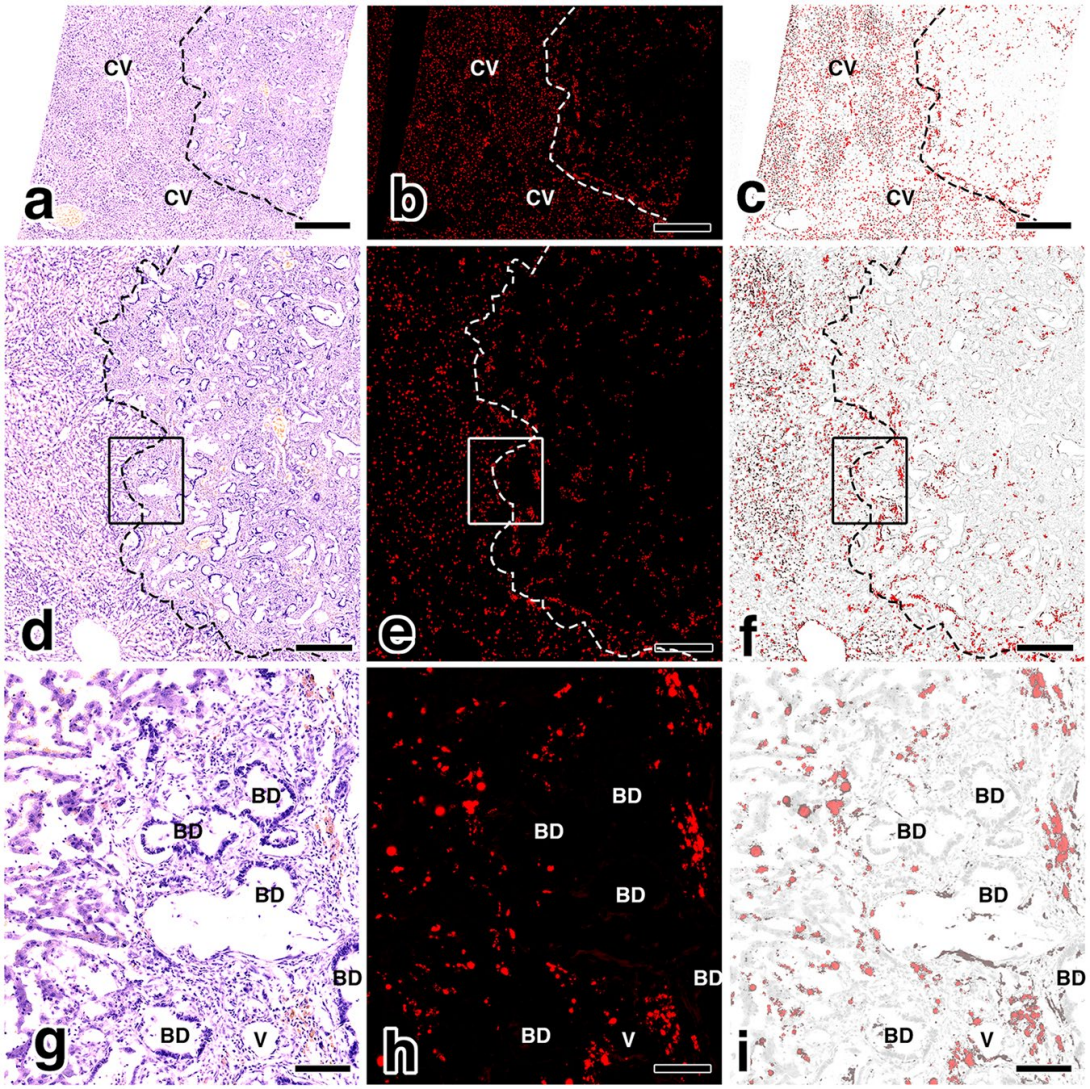
Histological FI of the pathological liver of the LEC rat. The HE section demonstrated liver lobules containing cellular plates were directed from the periphery of the liver lobules to the central veins (CV) on the left side of the slide at lower magnification (**a**). On the right side, there was hyperplasia of the ductules surrounded by fibrous tissue, indicating cholangiofibrosis (**a**,**d** and **g**). There were a larger number of labeled cells in the normal area on the left side than in the cholangiofibrosis area on the right side (**b**). The merged findings revealed that the cholangiofibrosis area did not contain as many labeled cells as the normal area (**c**,**f** and **i**). At higher magnification of the areas marked with rectangles (**d**–**f**), some labeled cells were detected in the cholangiofibrosis area **(g**–**i**). The labeled cells were located near the vessels (V) but not near the bile ducts (BD) (**f** and **i**). Scale bars:  (**a**–**c**) 1 mm, (**d**–**f**) 0.5 mm, (**g**–**i**) 0.1 mm.

The microscopic findings of the liver of the LEC rats injected with thiol-OS-MNP/Rho were observed using a virtual slide system. As shown in Fig. 5, the HE section demonstrated that liver lobules containing cellular plates were directed from the periphery of the liver lobules to the central veins on the left side of the slide at lower magnification (Fig. 5a). On the right side, there was hyperplasia of ductules surrounded by fibrous tissue, indicating cholangiofibrosis (Fig. 5a,d and g). These fibrotic changes might contribute a heterogeneous distribution of the labelled cells. There were a larger number of labeled cells in the normal area on the left side than in the cholangiofibrosis area on the right side (Fig. 5b). The merged findings revealed that the cholangiofibrosis area did not contain as many labeled cells as compared with that of the normal area (Fig. 5c,f and i). At higher magnification, some labeled cells were detected in the cholangiofibrosis area (Fig. 5g–i). The labeled cells were located near vessels (V) but not near bile ducts (BD) (Fig. 5f and i). These findings indicated that the localization of labeled cells was related with vessels in the pathological region of the LEC rat liver, and agreed with the finding of the mesoscopic FI (Fig. 3d–f). Histological FI using thiol-OS-MNP/Rho demonstrated that the distribution of labeled cells was related with the pathological findings. In previous paper, we reported that heterogeneous uptakes of Kupffer cells in mouse liver for thiol-OS-MNP/Rho using anti-CD68 antibody^23^. Some labeled cells were immunostained with anti-CD68 antibody, but some not. And some CD68 positive cells did not show fluorescence of thiol-OS-MNP/Rho. Therefore it should be noted that non-labeled cells with thiol-OS-MNP/Rho would be considered for further study to understand the relation between macrophage and tumor tissue. In addition, further investigations were required to understand the cellular uptake behavior of labeled cells. The distribution and behavior such as uptake kinetics would provide further characteristics and specificity of labeled cells in pathological regions. Correlative light electron microscopy (CLEM) including intravital fluorescent imaging, 3-dimentional analysis with de-colorization and confocal microscopy, transmission and scanning electron microscopy would be useful for not only *in vitro* as reported previously^26, 27^ but also *in vivo* evaluation. CLEM for *in vivo* is under developing.

A pathological liver of an LEC rat has various pathological findings. Further correlative studies would provide more relations between the pathological findings and the distribution of labeled cells. The accumulations of correlation study between histological FI and mesoscopic multimodal imaging, and between mesoscopic multimodal imaging and MRI, might find a link between pathological diagnosis and MRI via mesoscopic imagings. These links would allow MRI pathological diagnosis without biopsy using thiol-OS-MNP/Rho.

In conclusions, thiol-OS-MNP/Rho demonstrated mesoscopic multimodal imaging of hepatic tumor successfully. The thiol-OS-MNP/Rho could enhanced tumor nodule in liver of LEC rat on *in vivo* MRI non-invasively and semi-quantitatively, and revealed abnormal fluorescent pattern of pathological liver on mesoscopic FI. Mesoscopic imaging of *ex vivo* pathological liver on MRI and FI revealed “differential” multimodal imaging from bright field image, and could provide additional information such as peripheral distribution of labeled cells in invisible pathological area on MRI. Histological FI revealed the distribution of labeled cells associated with pathological findings, and relations with mesoscopic FI. These results demonstrated that imaging using thiol-OS-MNP/Rho provided seamless and sequential “identical” multimodal imaging of pathological tissue. In addition, we demonstrated the difference between the findings of bright field imaging and that of MRI. We would like to emphasize that not only “identical” but also “differential” multimodal imaging are very useful to evaluate and understand pathological regions. Multimodal imaging including mesoscopic “macrophage imaging” in liver would provide new insight and have great possibilities to develop advanced imaging and innovative nanomedicine.

## Methods

### Materials

MPMS and rhodamine B were purchased from Sigma-Aldrich Chemical co, (St. Louis, MO). MNPs EMG606 was from Ferrotec corp. (Tokyo, Japan). Ethyl alcohol and 28% NH_4_OH from Wako Fine Chemicals Inc. (Osaka, Japan).

### Preparation of thiol-organosilica nanoparticles containing MNP and Rhodamine B

The mixture of 12.5 mM MPMS, 0.1% EMG707, 2.0 mM rhodamine B, and 27% NH_4_OH, was incubated to grow the organosilica layer as reported previously^23^. After incubation, the reaction mixture was subjected to centrifugation to remove remaining reagents, and the pellet was sonicated. The particles were washed extensively with distilled water.

### Characterization of thiol-OS-MNP/Rho

Relaxation times (T1 and T2) of NPs were measured with a 3-T PET/MR equipment (Biograph mMR, Siemens Healthcare, Erlangen, Germany) with 12ch head coil. Phantom materials containing of various concentrations of thiol-OS-MNP/Rho (0, 1.56 3.12, 6.25, 12.5, 25, and 50 µg/mL) with 1.5% agar were prepared. T1 relaxation time of the phantom was calculated using the images obtained with a number of turbo inversion recovery sequences with different inversion times (TI) of 100, 200, 400, 800, 1000, 1500, 2000, and 3000 ms, a repetition time (TR) of 3000 ms, echo time (TE) of 12 ms, 3 mm thickness, FOV 240 mm^2^, matrix of 192, and NEX 1. T2 relaxation time was also calculated using the images obtained with a turbo spin echo sequence having fixed TR of 4000 ms and various TE values of 13.3, 26.6, 39.9, 53.2, 66.5, 79.8, 93.1, and 106.4 ms, FOV 240 mm^2^, matrix of 192, and NEX 1. All analyses and calculations of relaxation times were performed with Image J (NIH, Bethesda, MD) and Microsoft Excel 2013. R1 and R2 were calculated using these calculated T1 and T2 values, respectively.

Fluorescence in a tube containing 0.1 mg/ml thiol-OS-MNP/Rho was observed with a multi-purpose zoom microscope (AZ100M, Nikon, Kanagawa, Japan) equipped with a 100-W mercury lamp as a light source and a CCD camera (Digital sight DS-Ri1, Nikon, Kanagawa, Japan). Excitation and emission wavelengths were chosen as 540 and 605 nm, respectively. The absorbance of the solution containing 0.05 mg/ml thiol-OS-MNP/Rho, 20 µM Rhodamine B, 0.05 mg/ml EMG707, and 0.05 mg/ml organosilica particles without rhodamine B nor EMG707 were measured with a V-730iRM spectrometer (JASCO, Tokyo, Japan). The fluorescent intensities of 2 µM Rhodamine B solution and 0.1 mg/ml thiol-OS-MNP/Rho solution were obtained with an F-4500 fluorescence spectrophotometer (Hitachi, Tokyo, Japan).

### MRI of LEC Rats Injected thiol-organosilica nanoparticles containing MNP and Rhodamine B

All experiments were performed in accordance with Tokushima University institution’s guidelines for animal care and use. All experimental protocols were approved by the animal research committee of Tokushima University. LEC rats (57 weeks) were injected intravenously with 0.9 mL of a solution containing 10 mg/mL of thiol-OS-MNP/Rho, respectively. After 1 day, these rats were examined on MRI. MRI images were performed using a 3-T Signa Excite HDxt (GE Healthcare, Milwaukee, WI, USA) with 8ch head coil. MRI pictures were reconstructed using OsiriX software (OsiriX Foundation, Geneva, Switzerland).

### Mesoscopic Fluorescent Imaging and Histological Observation of Livers of LEC Rats

The livers of LEC rats injected thiol-OS-MNP/Rho were observed by using multi-purpose zoom microscope. These livers were fixed with 4% paraformaldehyde in 0.1 mM phosphate buffer (pH 7.4) for 4 h and then rinsed sequentially with PBS containing 10, 15, and 20% sucrose for 4 h. The tissues thus fixed were placed in OCT compound, and frozen at −80 °C. Frozen sections (6-μm thickness) were prepared with a cryostat and mounted on glass slides. The section slides were scanned with a NanoZoomer HT slide scanner with NanoZoomer Digital Pathology Scanner System software (Hamamatsu Photonics KK, Shizuoka, Japan). After scans in fluorescence mode, the liver sections were stained with hematoxylin and eosin, and then were scanned in bright-field mode. The analysis of digital slides was preformed with NDP Viewer Dual (Nano Zoomer Digital Pathology, Hamamtsu Photonics KK, Shizuoka, Japan).

### Mesoscopic MRI of the Liver of LEC Rat

The fixed liver of LEC rat was sectioned into small piece. MRI images were performed using a MR VivoLVA (DS Pharma Biomedical Co., Ltd, Osaka, Japan). Three-dimensional MRI pictures were reconstructed using OsiriX software (OsiriX Foundation, Geneva, Switzerland).

## Electronic supplementary material

Transverse findings of T2-weighted MRI.

Transverse findings of T1-weighted MRI.

Three-dimensional video of a section LEC rat liver on T2-weighted MRI.

3-dimentional image of LEC rat liver on 3D-SPGA MRI

Mesoscopic fluorescent image movie of LEC rat liver
